# Parents' Stress and Children's Psychological Problems in Families Facing the COVID-19 Outbreak in Italy

**DOI:** 10.3389/fpsyg.2020.01713

**Published:** 2020-07-03

**Authors:** Maria Spinelli, Francesca Lionetti, Massimiliano Pastore, Mirco Fasolo

**Affiliations:** ^1^Department of Neurosciences, Imaging and Clinical Sciences, University G. D'Annunzio Chieti-Pescara, Chieti, Italy; ^2^Department of Biological and Experimental Psychology, Queen Mary University of London, London, United Kingdom; ^3^Department of Developmental Psychology and Socialisation, University of Padua, Padova, Italy

**Keywords:** COVID-19, parents, children, parent stress, children behavioral problems, children emotional problems

## Abstract

**Objectives:** The present study aimed to explore the effect of risk factors associated with the COVID-19 outbreak experience on parents' and children's well-being.

**Methods:** Parents of children aged between 2- and 14-years-old completed an online survey reporting their home environment conditions, any relation they had to the pandemic consequences, their difficulties experienced due to the quarantine, their perception of individual and parent-child dyadic stress, and their children's emotional and behavioral problems.

**Results:** Results showed that the perception of the difficulty of quarantine is a crucial factor that undermines both parents' and children's well-being. Quarantine's impact on children's behavioral and emotional problems is mediated by parent's individual and dyadic stress, with a stronger effect from the latter. Parents who reported more difficulties in dealing with quarantine show more stress. This, in turn, increases the children's problems. Living in a more at-risk area, the quality of the home environment, or the relation they have with the pandemic consequences, do not have an effect on families' well-being.

**Conclusions:** Dealing with quarantine is a particularly stressful experience for parents who must balance personal life, work, and raising children, being left alone without other resources. This situation puts parents at a higher risk of experiencing distress, potentially impairing their ability to be supportive caregivers. The lack of support these children receive in such a difficult moment may be the reason for their more pronounced psychological symptoms. Policies should take into consideration the implications of the lockdown for families' mental health, and supportive interventions for the immediate and for the future should be promoted.

## Introduction

On 30th of January 2020, WHO declared a Public Health Emergency of International Concern after the first clusters of people infected by COVID-19 were diagnosed in China (WHO, 2020). The day after, the Italian Government started to define the first containment measures, such as checking people entering the country from China, in order to prevent the expansion of the contagion in the country (Government, 2020). However, from the second half of February the number of Italian cases increased, especially in Northern Italy. This led the Government to announce on February 21st the first restrictive measures in what was defined as the first Red Zone, including defined territories in the regions of Lombardia and Veneto, the areas most affected by the infection. Since the pandemic kept spreading around the country, the Prime Minister issued on March 9th a decree which extended to the entire national territory the restrictions already in force locally. The rules were supposed to last until April 3rd, but were extended by two more decrees firstly until April 13th and, later, until May 3rd (Government, 2020). At the time of writing (April 26th, 2020), there were in Italy 199,000 confirmed cases and 26,977 deaths, more than half of which occurred only in Lombardia and Veneto. When the data of the present study were collected (between the 2nd and the 7th of April), those numbers were still increasing, showing that the end of the pandemic is still a long way off.

The measures, known as #Iamstayingathome (#IoRestoaCasa), include the closure of shops, except those selling crucial necessities, the cancellation of all sports events, and the shutdown of schools and universities across the country (Government, 2020). With schools, all the educative supporting services directed to children of all ages were closed, with teachers from primary grade onwards providing online lectures. Quarantine began for the entire population; everyone was banned from leaving home except for non-deferrable and proven work or health reasons, or other urgent matters. Smart working has been incentivized, but since most activities are closed many people lost their job or went through a severe reduction of their income.

The life condition of families suddenly and deeply changed. In the home environment, the educational role of parents for children has become even much crucial than before. Children have only their parents around them, to provide support with homework when necessary and promote a positive development and new learning experiences for toddlers and preschoolers (Wang et al., 2020). Parents have been left alone not only in taking care of home-schooling their children, but also in general in the management of their children and of the home environment. All other educational services are closed, babysitters and grandparents are not available, and contact with peers is not allowed. Many parents also must do smart-working, and handling time and spaces to work with children around may be very problematic. Though quarantine means that time that can be shared with loved ones has increased, it also poses a major burden on parents' shoulders, as they are called to take an educational role while also trying to live their own lives and get on with their everyday job commitments. This situation has significantly increased the risk of experiencing stress and negative emotions in parents, with a potentially cascading effect on children's wellbeing (Sprang and Silman, 2013).

Hence, despite its positive effect in reducing the number of new infected cases, the mobility restriction and social isolation associated with quarantine are major concerns for families' psychological wellbeing. Related to this, the health care situation of the country is fragile, calling for attention. Hospitals are overcrowded, and the number of deaths is still increasing, as well as the number of infected people and those recovering in hospitals (Government, 2020). It is becoming very common to know at least one person who tested positive to COVID-19 or was hospitalized, and, most regretfully, to have experienced the loss of a person due to COVID-19. This might generate fear and preoccupation in parents and children, even for families who do not have to face health problems (Liu et al., 2020). Literature concerning previous experiences all over the world that may have some aspects in common with the COVID-19 situation reported a high presence of psychological distress such as depression, stress, irritability, and post-traumatic stress symptoms associated with quarantine (Hawryluck et al., 2004; Brooks et al., 2020) with long-lasting effects continuing for years after the event (Liu et al., 2012).

The majority of studies conducted during previous pandemics and from the beginning of the COVID-19 outbreak examined psychological consequences on the general population, leaving the study of effects on parents and children mainly unexplored, with few exceptions (Brooks et al., 2020). One study found that levels of post-traumatic stress were four times higher in children who had been quarantined than in those who were not (Sprang and Silman, 2013). A preliminary study conducted in China reported the presence of psychological difficulties in children during the COVID-19 pandemic, with fear, clinging, inattention, and irritability as the most severe symptoms for younger children (Jiao et al., 2020). Still, mechanisms that might explain what specific COVID-19 related risk factors put children more at risk of negative outcomes, and what is the interplay between COVID-19 lockdown and parents' wellbeing on children's adjustment, have not been investigated yet. A deeper understanding of family processes, protective factors, and risk factors in the home environment might be important if the wellbeing of children is to be promoted in these difficult times (Wang et al., 2020).

The present study wants to shed light on families' well-being during the COVID-19 outbreak in Italy, by exploring parents' and children's individual and dyadic adjustment after one month of quarantine. Understanding parents' and children's reactions and emotions, and identifying risk and protective factors, is essential to properly address their needs to tailor present and future intervention programs (Sprang and Silman, 2013).

In general, little is known about which factors may be associated with protection against child behavioral and emotional problems during a health emergency. In order to fill this gap, the main aim of the present study was to explore how pandemic-related variables, structural aspects of the home and family environment, and parental subjective experience of stress and adjustment to the quarantine, affect the wellbeing of parents and children, and how in turn the well-being of parents and children are associated. Specifically, we explored both individual parent stress and dyadic perception of stress since it is well-know that both levels of stress may impair children's well-being (Belsky, 1984; Abidin, 1992; Madigan et al., 2018; Martin et al., 2019). We expected that implications of the COVID-19 outbreak might increase parents' psychological difficulties, particularly stress both at the individual and the dyadic level, with a consequent negative impact on children's emotional and behavioral well-being (Dalton et al., 2020).

## Methods

### Study Design and Participants

Parents filled out an anonymous online survey, after reading the written consent form and explicitly agreeing to take part in the study. The survey was shared via social media for a limited time (from April 2nd to 7th, 2020), targeting parents of children aged 2- to 14-years-old. In the case of multiple children, the parent was asked to report on one child only. All the questionnaires, both parent- and child-related, were completed by the parent. There was no monetary compensation for participating. The final sample providing information on all study variables consisted of 854 parents living in Italy, of which 797 were mothers (*M*age = 38.96(6.02) (49% of whom had a high school degree or less, 37% a bachelor's or master degree, and 21% a higher education degree) and 57 were fathers (*M*age = 41.9(6.75) (41% of whom had a high school degree or less, 33% a bachelor's or master degree, and 26% a higher education degree). Children's mean age was 7.14 (3.38); 427 were boys. A total of 271 parents were resident in the north of Italy where most COVID-19 cases, were registered i.e., Lombardia and Veneto (from now on defined as the Red Area). Data reported in this study are part of a wider longitudinal research project designed with multiple purposes related to the investigation of the psychological impact of the COVID-19 outbreak in Italian parents and children. The study was approved by the ethical commitment of the Department and was conducted according to the American Psychological Association guidelines in accordance with the 1964 Helsinki Declaration.

### Measures

#### COVID-Contact Risk Index

An *ad-hoc* index was computed to evaluate the amount of contact the parent had with people directly affected by the virus, following the assumption that the greater the number of contacts, and the closer the people affected by COVID-19 that the parent knows are to the parent, the greater the impact on psychological wellbeing would be. One point was given for each of the following if present: the parents tested positive for the virus, a familiar or close friend tested positive, a familiar/close friend was hospitalized, a familiar/close friend died. A half=point each was given if the parent knew a person (not familiar or close friend) who tested positive, was hospitalized, or died.

#### Home Environment Risk Index

An *ad-hoc* risk index was computed to evaluate the house and family situation, including factors supposed to be related to the quality of life condition. One point was given for each of the following: loss of job due to the pandemic, absence of external spaces (balcony or garden), total family income less than 1250 € per month, only one adult in the house in charge of the child, no Wi-Fi, no pets. To compute the index, this score was summed with the number of rooms/number of people ratio in the house.

#### Quarantine Parent Risk Index

Difficulties experienced by parents during the quarantine were investigated with a newly developed pool of 13 items. Parents were asked to indicate, using a 7-point Likert scale, how difficult they were perceiving, during the last week, dealing with several aspects related to the quarantine such as finding a relaxing space alone to unplug, time for the partner and for kids, and to do activities such as sport, reading, cooking, etc. (see Appendix 1 for the full list of items). Cronbach's alpha was 0.84, with 95% CIs [0.83–0.84].

#### Parent's Dyadic Parenting Stress

Perception of parent's stress in the parent-child interaction was investigated using the 15 items Parent/Child Dysfunctional interaction domain of the Parenting-Stress Index Short Form (PSI) (Abidin, 1995). The scale investigates with a 5-point rating scale the extent of parents' agreement or disagreement with statements describing the parent–child relationship as difficult to manage. Cronbach's alpha in the current study was 0.86, 95% CIs [0.86–0.86].

#### Parent's Individual Stress

Parent's individual perception of stress was investigated using the 7 items from the Stress subscale of the Depression Anxiety Stress Scale–Short form (DASS) (Lovibond and Lovibond, 1995). The scale provides on a 5-point rating scale a measure of individual symptoms indicating stress i.e., irritation and agitation. To obtain the total score, items are summed. Cronbach's alpha in the current study was 0.88, 90% CIs [0.88–0.89].

#### Children's Psychological Problems

Behavioral and psychological problems in children were investigated using the parent-report form of the Strengths and Difficulties Questionnaire (SDQ) (Goodman, 2001). The current study focuses specifically on the following subscales: emotional symptoms, hyperactivity-inattention, and conduct problems. Each subscale is measured by 5 items, rated on a 3-point scale. To obtain the total scores, items are summed. Cronbach's alpha in the current study were as follow: 0.64 for the emotional symptoms scale (90% CIs [0.62–0.66]), 0.73 for the hyperactivity-inattention scale (90% CIs [0.72–0.75]), and 0.53 (90% CIs [0.51–0.55]) for the conduct problems scale. Values were comparable to those reported in the Italian evaluation of the SDQ (Tobia and Marzocchi, 2018).

### Analytic Plan

First, descriptive statistics and bivariate correlations among study variables were presented. Afterwards, two multivariate mediation models were tested, including as a predictor relevant quarantine-related risk factors (derived from the correlational analysis), as a mediator parents' stress (in one model dyadic parenting stress was explored as the candidate mediator, in the other model it was individual stress) and as outcomes children's psychological problems at the SDQ. Mediation models were compared with a with a null model and a main effect model, including only quarantine-related risk factors as the predictor. Akaike weights, providing the probability of a model to support new data conditional on the set of models considered, were used for model comparison (Wagenmakers and Farrell, 2004). Parameters were investigated for the best fitting model. Finally, as a follow-up analysis, we explored whether results were comparable distinguishing between parents' living in the Red Area (including Lombardia and Veneto regions) with the rest of the sample. To this aim, we performed a multi-group analysis. Analyses were run using the statistical software R (Team, 2018), lavaan package (Rosseel, 2012). Plots were depicted using package ggplot2.

## Results

### Descriptive Statistics

Means, SDs, and correlation values among variables of interest are reported in Table 1. Due to the large sample size, correlation values above 0.06 (i.e., trivial in effect size) were significant at *p* < 0.05; thus, for interpreting effects, we considered the strength of the association (namely Pearson's *r*) as an effect size. Results showed that overall there were no relevant associations of COVID-contact risk index and Home environment risk index with dyadic parenting stress (PSI), parent's individual stress (DASS), and children's psychological problems (SDQ).

**Table 1:** Descriptive and bivariate correlations.

	**Mean (SD)**	**1**	**2**	**3**	**4**	**5**	**6**	**7**	**8**	**9**
1. COVID-RI	0.33 (0.54)									
2. Home-RI	1.85 (0.91)	−0.05								
3. Quarantine parent-RI	46.29 (16.02)	0.07	−0.04							
4. Red Area		0.15^**^	−0.03	0.10^*^						
5. Child age	7.14 (3.83)	0.02	0.03	−0.22^**^	0.03					
6. PSI stress	22.01 (7.65)	−0.02	0.10^*^	0.20^**^	0.00	0.13^**^				
7. DASS stress	29.57 (10.28)	0.07^*^	0.05	0.36^**^	0.03	−0.08^*^	0.41^**^			
8. SDQ emotional symptoms	7.09 (1.84)	−0.01	0.02	0.17^**^	0.05	0.13	0.39^**^	0.32^**^		
9. SDQ Hyper.–inattention	8.90 (2.31)	−0.06	0.09^*^	0.22^**^	0.03	−0.21	0.44^**^	0.32^**^	0.32^**^	
10. SDQ conduct problems	7.23 (1.56)	−0.05	0.10^*^	0.23^**^	0.01	−0.05	0.47^**^	0.33^**^	0.38^**^	0.54^**^

*RI, Risk Index; Red Area: 1, Lombardia or Veneto; 0, all other regions. PSI stress, Dyadic parenting stress in the child-parent interaction as from the Parenting Stress index; DASS stress, Individual stress as from the Depression Anxiety Stress scale; SDQ, Strengths and difficulties questionnaire*.

* *p < 0.05*,

** *p < 0.001*.

### Multivariate Regression Models

Because the only risk factor associated with parent's individual and dyadic stress and children's psychological problems was the Quarantine parent risk index, we did not include in the model the Home and COVID risk indices. Thus, models tested had as a predictor the Quarantine parent risk index, as the candidate mediator parent stress (dyadic and individual), and as outcomes children's emotional and behavioral problems.

**Figure 1:**
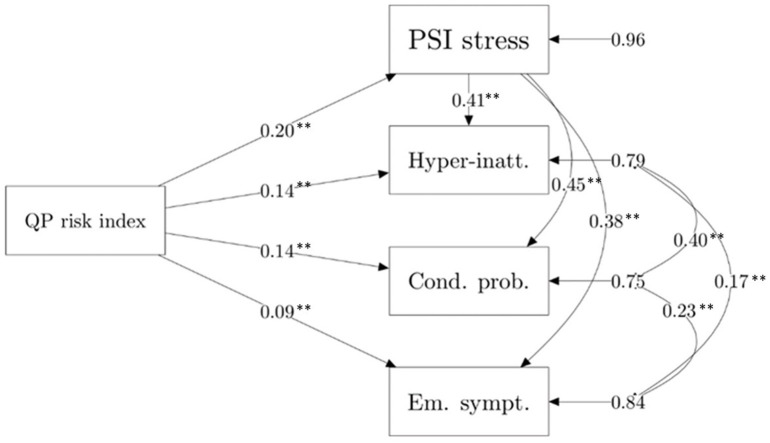
Multivariate mediation model, including dyadic parenting stress (PSI stress) as a mediator. QP risk index, Quarantine parent risk index. **p < 0.01.

**Figure 2:**
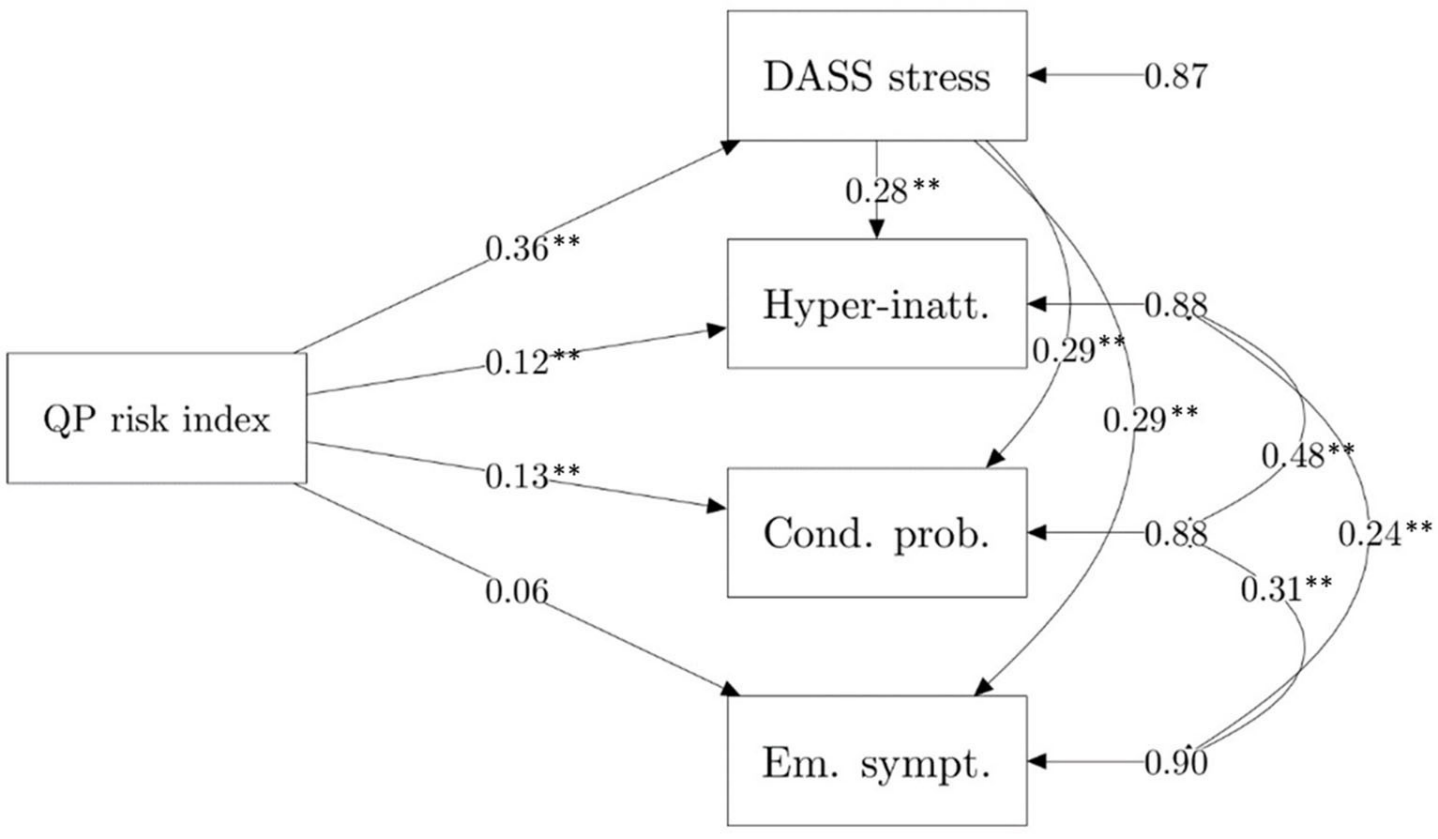
Multivariate mediation model, including individual parent stress (DASS stress) as a mediator. QP risk index, Quarantine parent risk index. **p < 0.01.

For both the model including dyadic parenting stress as a mediator and individual stress as a mediator, the mediation model outperformed the null and main-effect regression model. Specifically, for the model including dyadic parenting stress as a mediator, Akaike weights were lower than 0.001 for both the null and the main effect model, and very close to 1.00 for the mediation model. The same weights were obtained for the comparison with the mediation model including individual stress. Standardized estimates of the two mediation models are reported in Figures 1, 2. Parameters for indirect effects and proportion of variance explained for each outcome variable for the investigated models are reported in Table 2.

**Table 2:** Defined parameters.

	**Dyadic parenting stress as mediator**			**Individual stress as mediator**		
	**ß**	***p***	***R*-square**	**ß**	***p***	***R*-square**
Emotional symptoms			0.163			0.102
Indirect effect	0.074	<0.001		0.106	<0.001	
Total effect	0.167	<0.001		0.167	<0.001	
Conduct problems			0.245			0.124
Indirect effect	0.088	<0.001		0.103	<0.001	
Total effect	0.231	<0.001		0.231	<0.001	
Hyperactivity-inattention			0.214			0.117
Indirect effect	0.082	<0.001		0.100	<0.001	
Total effect	0.225	<0.001		0.225	<0.001	

### Follow-Up Analyses

Because of the significant association between study variables and age, we ran the analyses again, including the effect of the child's age on the mediator and outcome variables. Results remained stable overall. With a multi-group analysis, we finally explored whether results were comparable for residents in the Red Area (Lombardia and Veneto) vs. other regions. No relevant differences were identified. Results are available upon request to the corresponding author.

## Discussion

The COVID-19 outbreak is a completely new and unexpected situation currently affecting many countries. Italy was, after China, the second most highly affected country at the time, with the pandemic spreading very fast. In just a few weeks, the population found itself from thinking that the pandemic was happening far away, to being directly involved (Government, 2020). The closure of schools and the decision to keep children locked at home was obvious, but the consequences of all this for families' well-being were barely considered.

Our study is the first to examine the impact of the COVID-19 outbreak on parents' and children's wellbeing. We explored bivariate associations among the environment, family, and COVID-19 outbreak-related factors on parents' stress and children's psychological problems, and the interplay among these variables. Results showed that factors such as living in a more at-risk contagion zone or being in closer contact with the virus' effects do not relevantly affect parents' and children's well-being. This confirms findings from a preliminary study in China, where the difference in children's symptoms between areas identified by different levels of epidemic risk was not statistically significant (Jiao et al., 2020). Similarly, the quality of the environment, such as the physical characteristics of the living space, is not associated with parents' and children's psychological symptoms. Yet, it is the parents' individual perception of the situation, and more specifically how difficult they find it dealing with the many stresses the quarantine imposes, that is significantly associated with parent's stress and children's psychological problems, and that indirectly impacts on children's behavioral and emotional problems through the mediating role of parent's stress. Parents who report finding taking care of their children's learning, finding space and time for themselves, the partner, the children, and for the activities they used to do before the lockdown more difficult, are more stressed. This confirms studies that found an effect of the limitations associated with quarantine on the well-being of adults (Brooks et al., 2020). We further add to the literature that this stress is experienced both at the individual (e.g., being over-reactive, feeling nervous and irritated) and at the dyadic level (e.g., finding it difficult to enjoy interactions with the child, and child behavioral and emotional expressions). In addition, we pointed out that it is this stress that significantly impacts on children's well-being. Hence, it is mainly when the strains of quarantine affect the ability of the parent to enjoy and appreciate the parent-child relational experience that the consequential negative impact on the child's well-being is stronger, a result with important implications for informing intervention programs that target the family and the child. Moreover, this impact is present at every age, even though our age range is quite wide. This underlines that the impact of the lockdown on parents and children is present with similar mechanisms for families with children younger than 14 years.

The effect we identified in our study may be explained in many ways. More stressed parents find it more difficult to understand their child's needs and to respond in a sensitive way (Abidin, 1992; Scaramella et al., 2008). Stress is often associated with rude behaviors and difficulties in explaining limits and discipline. Thus, children in these families may feel less understood by their parents and may react in more negative and aggressive ways (Pinquart, 2017). Moreover, we know that children have lower personal resources to deal with the many changes the pandemic is imposing on their life (Liu et al., 2020) and guidelines suggest parents should discuss and explain the situation with them, since correct information about what is happening and the reasons for the restrictions children have to face is crucial to prevent negative psychological consequences (Dalton et al., 2020). However, how and when to do that is completely left up to the parents' choice. We can speculate that more stressed parents may be too overwhelmed by the situation to find appropriate ways to be a supportive figure for their children and to find the best ways to address children's questions and fears (DiGiovanni et al., 2004). When children do not find responsive answers to their preoccupations from adults, they may show more distress, evidenced by more emotional and behavioral problems as well as inattention and difficulties in concentrating.

These results suggest many interesting implications that should be addressed in the present and in the future in Italy, and in all countries involved in the pandemic, if we want to promote children's wellbeing, and prevent the onset of more severe behavioral and emotional problems. The pandemic and the quarantine associated with it require using personal resources to deal with everyday life and fears and worries. Correct information and guidelines have to be given to adults about how this stressful situation may affect their personal and children's wellbeing. Public health should provide parents with knowledge about, for instance, how children at different ages express distress and the importance of sharing and talking about fears and negative emotions (Dalton et al., 2020). In this way even less resilient and more stressed parents may be helped in finding ways to understand and support their children (Belsky, 1984).

The closure of schools may have also contributed to this phenomenon. Firstly, because parents are left alone dealing with their children's education and learning, this may be a very challenging duty. Moreover, teachers have a role not only in delivering educational materials but also in offering an opportunity for children to interact, and to receive from them support and explanations. Organizing online courses in a way to also improve the possibility for children to interact with their teacher about things outside of the learning context should be a priority especially if school closures are to be prolonged. Moreover, the Government should take into consideration the impact of school closures on parents by finding ways to help them deal with the learning experience of children and with having children at home 24/7, while parents also have to manage home-working and childcare. This is going to be even more relevant if, during the second phase of the emergency, job activities will re-open, and parents will be asked to go back to work, but schools will be kept closed. How are parents supposed to deal with this?

Some limitations of the present study should be addressed. Firstly, this is a correlational study; a longitudinal exploration of the effects of quarantine on parents and the cascading effects on children over time would help in better understanding the phenomenon. Moreover, we have collected children's psychological symptoms from parent reports; although this data collection method is widely used it may be less informant than child reports or direct evaluation of children's well-being made by experts. Lastly, we may expect that quarantine risk is higher for more at-risk families i.e., families of separated parents, families with children with disabilities, very poor families, etc. The exploration of the phenomenon with those in at-risk situations would help in developing more tailored interventions.

If properly supported by healthcare professionals and other social connections, including the school environment, parents and children can appropriately overcome this critical period of distress and avoid severe long-term consequences. Quarantine and social distancing are efficient ways to deal with the pandemic, but these experiences may have consequences on people's well-being. However, the media and public institutions concentrate primarily on physical health to recommend steps for the prevention and containment of the disease, leaving the impact on mental health undiscussed. Indeed, stable mental health is one of the keys to fight this ongoing pandemic and to restore a post-pandemic society; the well-being of parents and children must be under surveillance since problems on this side may have long-lasting implications.

As Bowlby suggested 30 years ago, “Man and woman power devoted to the production of material goods counts a plus in all our economic indices. Man and woman power devoted to the production of happy, healthy, and self-reliant children in their own homes does not count at all. We have created a topsy-turvy world” (Bowlby, 1988).

## Data Availability Statement

The raw data supporting the conclusions of this article will be made available by the authors, without undue reservation.

## Ethics Statement

The studies involving human participants were reviewed and approved by Department of Neuroscience, Imaging and Clinical Sciences. The patients/participants provided their written informed consent to participate in this study.

## Author Contributions

MS, FL, and MF conceptualized the study and organized the data collection. MS and FL wrote the first draft of the manuscript. FL and MP run the analyses and wrote the results section. All authors contributed to revision of the final version of the manuscript.

## Conflict of Interest

The authors declare that the research was conducted in the absence of any commercial or financial relationships that could be construed as a potential conflict of interest.

## Abbreviations

SDQ: Strengths and Difficulties Questionnaire
PSI: Parenting Stress Index Short form.
